# Attitudes toward and Knowledge about Wolves in SW German Secondary School Pupils from within and outside an Area Occupied by Wolves (*Canis lupus*)

**DOI:** 10.3390/ani10040607

**Published:** 2020-04-02

**Authors:** Christoph Randler, Annkathrin Wagner, Alena Rögele, Eberhard Hummel, Iztok Tomažič

**Affiliations:** 1 Didaktik der Biologie, Department of Biology, Eberhard Karls University of Tuebingen, Auf der Morgenstelle 24, D-72076 Tübingen, Germany; ak.wagner.aw@googlemail.com (A.W.); alena.roegele@uni-tuebingen.de (A.R.); 2 Staatliches Seminar für Ausbildung und Fortbildung der Lehrkräfte Ludwigsburg, Königsallee 54-56, 71638 Ludwigsburg, Germany; Hummel.Eberhard@rs-lb.sem-bw.org; 3 Univerza v Ljubljani/University of Ljubljana, Biotechnical faculty, Večna pot 111, 1000 Ljubljana, Slovenija; iztok.tomazic@googlemail.com

**Keywords:** attitudes, wolf (*Canis lupus*), secondary school students, knowledge, conservation, fear

## Abstract

**Simple Summary:**

The wolf (*Canis lupus*) was extinct from large parts of Europe, but during the last decades, wolves re-entered their previous distribution area in Germany. The federal state of Baden-Württemberg has delineated a wolf area where some individuals are roaming around. We compared secondary school students from within and outside the wolf area, and analysed gender, age, and residency effects on attitude and knowledge. A total of 254 students from secondary schools participated in this study with a mean age of 12.63 ± 2.17. We asked for basic attitudes toward wolves and for knowledge about wolves. In detail, age was related to the subscale interest to learn, with lower interest scores related to an increasing age. Girls reported a higher level of fear of wolves, and, concerning residency, conservation attitudes were lower within the wolf area compared to outside. Boys had a higher level of knowledge than girls. A higher level of knowledge was related to a greater conservation attitude, a greater interest to learn, a lower level of fear/harm, and a lower acceptance of hunting.

**Abstract:**

Wolves (*Canis lupus*) were exterminated from most areas of western Europe during the last two centuries, but, during the last decades, wolves re-entered their previous distribution area in Germany. We compared secondary school students from within and outside a delineated wolf area, and analysed gender, age, and residency. A total of 254 students participated in this study (age: *M* = 12.63 ± 2.17). We used a measurement introduced which consisted of three parts, demographics, attitudes and knowledge. There was a significant overall effect of age, gender, and residency in attitudes toward wolves. More specifically, age was related to the subscale interest to learn, with lower interest scores related to an increasing age. Girls reported a higher level of fear. Conservation was lower within the wolf area than outside. Boys had a higher level of knowledge than girls. A higher level of knowledge was related to greater conservation, a greater interest to learn, a lower level of fear, and a lower acceptance of hunting. Hence, in order to improve students’ conservation attitudes, it would be useful to foster learning about wolves at school. Special attention should be paid to ensuring that girls also internalize the content of these lessons.

## 1. Introduction

In European countries, roughly one-third of mainland Europe hosts at least one large carnivore species. The abundance of large carnivores is stable or increasing, mainly due to protective legislation, supportive public opinion, and a variety of practices that allow coexistence between carnivores and humans [1]. The grey wolf (*Canis lupus*) is one of the most controversial predators in Europe and its acceptance is on average lower compared to bears [2]. Wolves were exterminated from most areas of northern and western Europe during the last two centuries [3]. Therefore, grey wolves are classified as an endangered species [4]. During the last decades, wolves re-entered their previous distribution area in Europe and Germany. As wolves can possibly attack livestock, this led to conflicts with stakeholders, such as shepherds [5]. In Germany, the first reproductive success of a resident pack of wolves was registered in Saxony in 2000 and the number of wolves in Germany has been growing ever since [6]. In total, there were about 105 wolf packs, 25 pairs, and 13 single individuals resident in Germany during the monitoring year in 2018/2019 [7]. In southwestern (SW) Germany, the federal state of Baden-Württemberg has delineated a wolf area where some individuals are roaming around. Some sheep killing is attributed to these wolves. The occurrence of wolves and the probable killing of sheep is regularly discussed in local newspapers. To check on possible differences concerning attitudes toward and knowledge about wolves, we intended to sample school students from within and outside this specific wolf area.

All large carnivores are the cause of potential and actual human–wildlife conflicts, and therefore can be endangered in nature by natural and human caused factors. Redpath et al. [8] wrote: “*finding effective ways of conserving large carnivores is widely recognized as a priority in conservation*” (p. 1). In general, 3500 respondents (age ≥15 years) in a Norwegian sample showed positive attitudes toward large carnivores (wolf, bear, lynx, and wolverine) [9]. The same applied for the acceptance of large carnivores. However, it was also found that although attitudes were generally positive, they differed between different groups of participants: negative attitudes increased with age and more fearful individuals or people living in smaller communities expressed more negative attitudes. People living within large carnivore areas and hunters also reported more negative attitudes. Moreover, men showed more positive attitudes. A recent meta-analysis [2] in Europe showed that people had more positive attitudes toward bears than toward wolves. While attitudes toward bears improved over time, attitudes toward wolves became less favorable [2]. Furthermore, younger and higher educated people had more positive attitudes toward wolves.

Previous research showed that attitudes toward wolves are determined by proximity and historical presence [10]. Although the general public stance toward wolf conservation is often positive, some differences can be detected for people living in wolf habitats [11,12,13]. Karlsson and Sjöström [14] reported that people living in wolf territories had more negative attitudes concerning the conservation of wolves than people living close to the wolf territories [14]. Similarly, the farther away the nearest wolf territory was, the more favorable the attitudes toward wolf conservation were [14]. Swedes living in areas where wolves have been restored, reported more negative attitudes than the general public [13]. Ericsson, and Heberlein [13] suggested that one negative event (either direct or indirect) may have an impact on attitudes, especially in people living in states with new or recovering wolf populations [15]. Roskraft et al. [9] showed that people are generally inclined to accept having large carnivores in their country, but not in their vicinity (>10 km). More direct experience with wolves also decreased their acceptance by the general public [16]. Taken together, these studies suggest a difference in attitudes between people living within and outside a nearly established or recovery wolf area. Furthermore, in longer established wolf areas, attitudes become more negative [2].

Studies about the general public or specific stakeholders, like hunters or livestock producers [17,18], have been carried out. However, primary or secondary school students are usually neglected [12], with some exceptions [19,20,21,22,23]. In a study of Prokop and Tunnicliffe [23], it was found that children at the age of 10–15 years are more familiar with unpopular animals, but their attitudes toward them are more negative compared to popular animals. Moreover, the study has shown that girls had more negative attitudes toward wolves compared to boys. On the one hand, this can be explained from an evolutionary perspective, where a lower physical condition/ability of females does not allow them to escape from a predator attack [24], but on the other hand, some differences can also be attributed to social factors (in Prokop and Tunnicliffe, [23]. Bjerke et al. [11] described younger, rural respondents (aged 9–13) as being scared by wolves and showing more negative attitudes, especially when living in a wolf area. Ambarli [20] studied 215 rural and 98 urban secondary school students concerning their attitudes toward bears (*Ursus arctos*). Both groups liked bears, but were also afraid of them and unsure about living together with these carnivores in the future [20]. Rural students were more interested to learn about bears and their conservation [20]. Moreover, urban students reported less contact with nature and showed less positive attitudes toward bears. In a comparison between Turkish and Slovakian children, Turkish children reported less drastic stories about wolves and their interest in wolves was significantly higher than Slovakian children’s, while their fear of wolves was lower [25]. This might be owed to the fact that wolves have a higher density in Slovakia.

In the particular case of Germany, there are few studies about the acceptance or attitudes toward wolves in children and adolescents. Hermann and Menzel [26] showed in secondary school students that Wildlife Value Orientations and threat perception were useful predictors of an intention to support the return of wolves. Since students are the decision makers of tomorrow, research concerning their attitudes is of high scientific and social relevance. The negative attitudes of students, which may also be due to a lack of knowledge about wolves, can have a concrete influence on the conservation of wolves later. During school time, this can be counteracted by specific information and education about wolves, so that the conservation of wolves in Germany can be further promoted. This study contributes to the little amount of available studies regarding the knowledge about and attitudes toward wolves in students.

Specifically, the study has three aims: first, to compare students from outside and within the wolf area, second, to assess gender effects, and, third, to check possible age effects, concerning both attitudes toward and knowledge about wolves.

## 2. Materials and Methods 

### 2.1. Wolf Area in Baden-Württemberg

**Figure A1:**
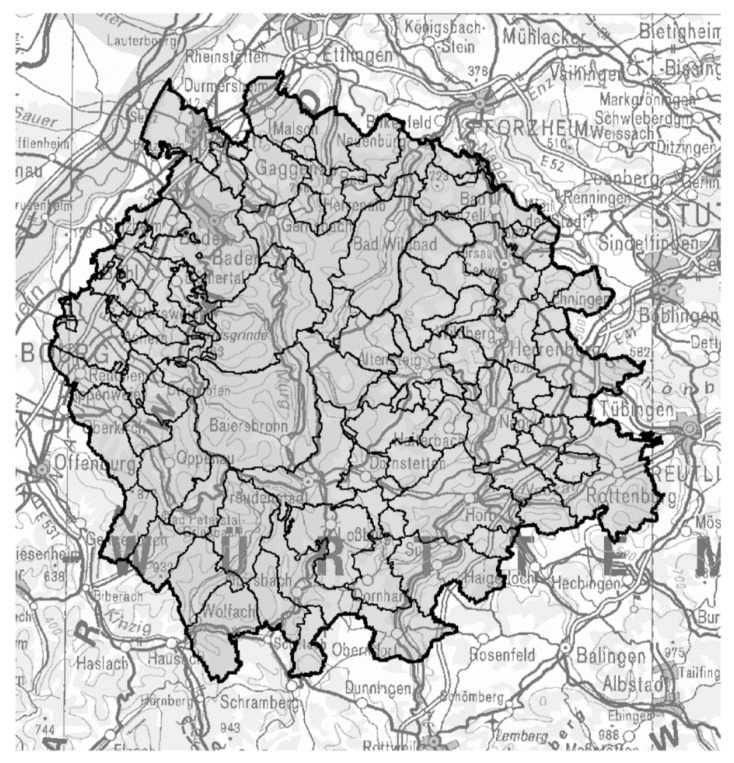
Wolf area in Baden-Württemberg (depicted in dark grey; Ministerium für Umwelt, Klima und Energiewirtschaft Baden-Württemberg, 2018 [39]).

Since 2015, a total of seven wolves have been clearly identified in Baden-Württemberg. Only one of them has settled in the northern Black Forest since 2017 [27]. The federal state government of Baden-Württemberg delineated an area with a radius of about 30 km around the permanent wolf territory, hereinafter referred to as “wolf area” [28]. A map of the wolf area in Baden-Württemberg is added as Appendix A Figure A1.

### 2.2. Sample

A total of 254 students from secondary schools participated in this study (104 boys, 147 girls, 3 divers). The mean age was 12.63 ± 2.17 (the range was 9–19 years). In total, 161 students were from within the wolf area, and 89 were from outside the area. We aimed at similar sample sizes, but the schools had the decision to participate or not, which makes it very difficult to achieve higher sample sizes. The study was approved by the higher authority (Ministerium für Kultus und Sport Baden-Württemberg, 31-6499.20/1307). 

### 2.3. Measurements

To assess the attitudes toward and knowledge about wolves, we used the measurement introduced by Oražem and Tomažič [19] and Oražem et al. [29]. The questionnaire administered to the respondents consisted of two parts, one part concerning attitudes and the second part assessing knowledge. It was developed at the Biotechnical Faculty in Ljubljana and it is based on items originating from Kellert’s [30] typology of basic attitudes. The first part was composed of 20 attitudinal items concerning wolves. A 5-point Likert type scale (from 1–strongly disagree to 5–strongly agree) was used to measure the respondents’ attitudes toward (a) the conservation of wolves, (b) fear/harm of wolves, (c) an interest to learn about wolves, and (d) an opposition to hunting or keeping wolves in captivity. High scores on the respective scales represent: greater conservation, a higher level of harm/fear, a greater interest to learn, and a greater acceptance of hunting. The part of the questionnaire concerning attitudes toward wolves is attached as Appendix A Table A1, with the wording of each item (in English and in German). The second part of the questionnaire assessed the students’ knowledge of wolves using 12 true/false statements and 10 multiple choice questions related to biology and wolf conservation. For each question, a “*Don’t know*” option was included in order to reduce guessing. For statistical analyses, the mean score of correct answers was calculated for every student, which ranges from 0 (no answers correct) to 1 (all answers correct). The original version was in Slovenian, which was translated and published in English [19]. The English version of the questionnaire was translated into German in a parallel analysis by three independent people. A fourth person was used as a judge to compare the translations and decide which version for every question should be used. Afterwards, the content of the questions was discussed with Iztok Tomažič who is fluent in English and Slovenian and with some basic knowledge of German. The questionnaire is presented in Appendix A Table A1.

### 2.4. Statistical Analyses

We applied a confirmatory factor analysis with AMOS 26 to assess the posited factor structure from Oražem and Tomažič [19]. Additionally, we used Cronbach’s α for internal consistency. A multivariate general linear model (GLM) was applied with the four subscales: (i) conservations, (ii) fear/harm, (iii) interest to learn, and (iv) hunting as dependent variables, and gender, age, and residency as independent variables. Subsequent univariate linear models were carried out to check every subscale separately. For the analysis of the knowledge scores, a univariate linear model was used with gender, age, and residency as independent factors. SPSS 26 was used for analyses. All linear models included the interaction terms by default. As these interactions were all not significant, we deleted them from the model and re-ran the analyses. We applied a confirmatory factor analyses with AMOS 26. The fit values were mediocre, especially the RMSEA, which was 0.088, with the 95% confidence interval between 0.079 and 0.097. Cronbach’s α was 0.74 for conservation, 0.83 for fear/harm, 0.84 for interest to learn, and 0.69 for hunting. These good reliability values suggest that the questionnaire can be applied in the German language as the original was Slovenian. Cronbach’s α of the knowledge scale was 0.65.

## 3. Results

The questionnaire showed good validity (see Section 2.4.) There was a significant overall effect of age, gender, and residency on attitudes toward wolves (Table 1). Therefore, we analysed the different scales separately to gain insight into the details of the results.

Based on the subsequent univariate analyses (Table 2), age was related to the subscale interest to learn, with lower interest scores related to an increasing age (r = −0.292, *p* < 0.001). Gender differences existed in harm/fear, with girls reporting a higher level of fear. As the number of diverse students was very small (N = 3), gender effects were only calculated for boys and girls. Concerning residency, there were no significant differences between the groups, except for conservation. The means for conservation were lower within the wolf area (4.14) compared to outside (4.39, *p* = 0.022).

With regard to knowledge, a univariate model was calculated with knowledge as a dependent variable and age, gender, and residency as independent variables (Table 3). Knowledge about wolves increased with age (r = 0.164, *p* = 0.009). Gender also revealed a significant effect. Boys had a higher level of knowledge than girls (0.56 versus 0.51).

Correlations between knowledge and attitudes are depicted in Table 4. A higher level of knowledge was related to a greater conservation attitude, a greater interest to learn, a lower level of fear/harm, and a lower acceptance of hunting.

## 4. Discussion

In this study, we compared secondary school students’ attitudes toward and knowledge about wolves within and outside a wolf area in SW Germany. We found effects from residency, age, and gender. 

First, we found a lower conservation attitude in students living within the wolf area compared to pupils outside. This corroborated by previous research, which associated the place of residence with attitudes toward wolves, mainly in adults [9,11,12,13,14]. Comparable to our results, the adult population also showed less positive attitudes when living in wolf regions [31]. Moreover, Bjerke et al. [11] reported adolescents showing more negative attitudes toward wolves when residing in a wolf area, where intense debates exist. In contrast, Oražem and Tomažič [19], working with a Slovenian adolescent sample, based on vocational high school students, reported no differences in attitudes. This is interesting because Oražem and Tomažič [19] used the same questionnaire and also conducted research with adolescents. One reason might be that there are many intensive discussions about wolves and their reoccupancy of SW Germany in social media and newspapers. Moreover, the wolves regularly kill sheep, and every case is discussed in the media, mainly because of compensation payments of stakeholders. This might explain the lower conservation attitude in the German sample. Furthermore, in contrast to Germany, wolves have never gone extinct in Slovenia. Therefore, wolves being quite a new occurrence in Germany might also lead to different attitudes compared to Slovenia. In the study by Oražem and Tomažič [19], students that are livestock breeders or have hunters in their families were equally distributed on the two groups of people living inside or outside a wolf area. As it is already known that the attitude of these interest groups can be less positive toward wolves than in the general public [9], the overall effect on attitude ratings was minimized due to the distribution of these students throughout the sample.

Concerning fear/harm, there were no differences between the areas, probably due to the low encounter rates of wolves with the public and the very low population size. In contrast to German adults [31], there were no differences in knowledge between residents in the wolf area and residents outside. This could be explained by the age of the respondents. In German adolescents, most knowledge may arise from school, while in adults, living in a wolf area might have an impact on knowledge, because adults acquire their knowledge by media news [31].

In this study, girls reported a higher level of fear compared to boys, which is corroborated by many other studies reporting higher levels of fear in adolescent girls [23,32] and/or adult women [12]. Oražem and Tomažič [19] found no gender differences in negativistic attitudes toward wolves, while Prokop and Tunnicliffe [23] reported similar results to ours with higher negativistic attitudes of girls toward wolves. Therefore, previous studies about gender differences regarding the fear of wolves could be replicated. As sample size and sampling procedure (questionnaire) were similar in Slovenia, it is surprising that there were no gender effects in the Slovenian sample. The Slovenian results are many times replicated in Slovenia, there were no differences in 7th graders [33], none in vocational students [19] and also no difference in fear for the Slovenian adolescent sample (general and vocational high schools; [29]). Wolves are probably more common in Slovenia but their representation is lower in the media. Similarly, in Turkey, Ambarli [20] found no gender differences in fear in secondary school students concerning bears. However, this might have been the case because Ambarli’s research [20] concerned bears and not wolves, and people generally show more positive attitudes toward bears than toward wolves [2]. Moreover, adolescents form Italy and Greece showed contradictory beliefs regarding wolves in a study by Hovardas and Korfiatis, which are influenced by inter-group aspects, such as inter-group relations [34]. This might also lead to different results in different samples, depending on the salient attitude.

Oražem and Tomažič [19] reported gender differences in the interest to learn (boys were less willing to learn) and acceptance of hunting, with boys showing a greater acceptance [19]. We were unable to find a greater acceptance of hunting in our sample, but in other German studies on animal welfare attitudes, boys expressed a higher acceptance of hunting compared to girls [35]. 

We found a higher level of knowledge score in boys compared to girls. This is remarkable, because girls perform better in biology, especially in species identification and ecology, than boys and have a higher level of knowledge [36]. Knowledge of wolves was not affected by gender in another study [23]. As fear often leads to avoidance of the feared subject, girls might try to avoid learning new information about wolves and tend to forget acquired knowledge quicker. Thus, girls reporting higher levels of fear in our study might also lead to these differences in knowledge between boys and girls. These results also fit in the discussion of gender differences from an evolutionary perspective of Prokop and Tunnicliffe [23], which is based on the idea that females would not have the physical ability to fight off a predator in the case of an attack [9], resulting in higher levels of fear towards predators. Therefore, females might avoid an encounter with wolves and possibly also further information concerning them. Further research is needed to check on this hypothesis. 

Usually, interest in species declines from the beginning of secondary education (grade 5 onwards, 10 years of age), and interest in biology declines significantly as well [36]. Similarly, animal welfare attitudes decline from grade 5 to grade 11 [35]. Losing interest in animals with an increasing age was also found in other studies for Slovenian [23,31,37,38] and German [36] students. This might be related to the beginning puberty and the growing interest in human anatomy/physiology or spending time with non-animal related hobbies or preparation of future careers [23].

Compared with Oražem and Tomažič [19], we found similar correlations in a similar strength, suggesting that the attitudes and the knowledge in Slovenian and German adolescents are somewhat comparable. In both countries, a higher level of knowledge was related to greater conservation attitudes. Inversely, a higher level of fear and a greater acceptance of hunting was related to lower knowledge scores. Similar to adolescents, a higher level of knowledge was positively related to greater tolerance in German adults [31]. Generally, it is difficult to assess in a cross-sectional design whether attitudes influence knowledge or vice versa.

The study provides new and valuable insight concerning students’ attitudes toward and knowledge about wolves. As German students have rarely been studied before, these data are of high importance for the conservation of wolves in Germany in the future. The results reveal a connection between knowledge and conservation attitudes, showing that students with greater knowledge have more positive attitudes. Hence, a misunderstanding of or misinformation about wolves might result in less conservation attitudes in students. Therefore, education at school should promote learning about wolves more strongly and focus on clearing up potential misunderstandings. As the value of wildlife and the perception of threat influence support for wolves in students [26], changes in attitudes toward wolves might directly have an impact on the conservation of wolves. In addition, schools should attach particular importance to impart knowledge about wolves to girls, as they know less about and are more afraid of wolves. In conclusion, there are simple ways to improve knowledge about wolves in students. These steps should be taken, as knowledge might influence attitudes, which lead to different actions. As students are the decision makers of tomorrow’s society, positive attitudes toward wolves are essential for further conservation of wolves in Germany.

## Figures and Tables

**Table 1:** Results of a multivariate general linear model with the four attitude subscales as dependent variables. The independent variables were age, gender, and residency. Partial Eta **^2^** can be understood as the explained variance.

	Wilks-Lambda	F	*p*	Partial Eta ^2^
Constant	0.110	473.176	<0.001	0.890
Residency	0.939	3.826	0.005	0.061
Age	0.794	1.554	0.021	0.056
Gender	0.902	6.362	<0.001	0.098

**Table 2:** Univariate analysis of the dependent variables (subscales) with residency, age, and gender as independent variables. Partial Eta **^2^** can be understood as the explained variance.

Source of Variance	Dependent Variable	Mean of Squares	F	Sig.	Partial Eta ^2^
Residency	Conservation	3.175	5.350	0.022	0.022
	Harm	1.650	1.797	0.181	0.007
	Learn	0.020	0.020	0.887	0.000
	Hunting	1.632	2.590	0.109	0.011
Age	Conservation	0.509	0.858	0.564	0.031
	Harm	1.014	1.103	0.361	0.040
	Learn	2.807	2.807	0.004	0.096
	Hunting	0.745	1.183	0.307	0.043
Gender	Conservation	0.000	0.000	0.989	0.000
	Harm	16.662	18.138	<0.001	0.071
	Learn	2.525	2.526	0.113	0.011
	Hunting	0.157	0.249	0.618	0.001

**Table 3:** Univariate model with knowledge scores as a dependent variable with age, gender, and residency as independent predictors. Partial Eta **^2^** can be understood as the explained variance.

Source	Mean of Squares	F	Sig.	Partial Eta ^2^
corrected model	0.053	2.489	0.006	0.103
Constant	14.821	700.635	0.000	0.746
Residency	0.031	1.469	0.227	0.006
Age	0.047	2.226	0.021	0.078
Gender	0.102	4.843	0.029	0.020

**Table 4:** Correlations between knowledge about and attitudes toward wolves. *** denotes *p* < 0.001.

		Knowledge
Conservation	Pearson’s correlation	0.320 ***
	Significance	<0.001
	N	254
Fear/Harm	Pearson’s correlation	−0.419 ***
	Significance	<0.001
	N	254
Interest to learn	Pearson’s correlation	0.257 ***
	Significance	<0.001
	N	254
Hunting	Pearson’s correlation	−0.277 ***
	Significance	<0.001
	N	254

## Appendix A

**Table A1:** Measurement for attitudes toward wolves.

Original Wording	German Wording
Conservation	
All wolves should be exterminated.	Alle Wölfe sollten ausgerottet werden.
There is no need to preserve wolves in Germany, because they live elsewhere in Europe.	Wölfe müssen in Deutschland nicht geschützt werden, weil sie auch anderswo in Europa leben.
In Germany, wolves should be preserved for future generations.	Wölfe sollten in Deutschland für zukünftige Generationen geschützt werden.
Wolves are evil by nature because they attack livestock (sheep).	Wölfe sind von Natur aus böse, weil sie Nutzvieh angreifen (Schafe).
If all wolves were killed in Germany, it would bother me.	Wenn alle Wölfe in Deutschland getötet würden, würde mich das stören.
Wolves should have rights too.	Wölfe sollten auch Rechte haben.
Fear/Harm	
I would be afraid walking through the forest, if I knew that wolves lived there.	Ich hätte Angst in den Wald zu gehen, wenn dort Wölfe leben.
I would camp only where there are no wolves.	Ich würde nur dort zelten, wo keine Wölfe sind.
I am afraid of wolves.	Ich habe Angst vor Wölfen.
Wolves should not be near human settlements.	Wölfe sollten nicht in der Nähe von menschlichen Siedlungen sein.
I would accept the wolf presence in forests near my neighbourhood.	Ich würde es akzeptieren, wenn Wölfe in benachbarten Wäldern leben würden.
Wolves are not dangerous to humans.	Wölfe sind für Menschen nicht gefährlich.
Interest to learn	
I would like to know how wolves developed.	Ich würde gerne wissen wie sich Wölfe entwickeln/aufwachsen.
I like to watch popular science braodcast about wolves.	Ich schaue gerne Tierdokumentationen über Wölfe an.
I would like to learn about different habitats of wolves.	Ich würde gerne etwas über die verschiedenen Lebensräume von Wölfen lernen.
I like to read about wolves.	Ich lese gerne etwas über Wölfe.
Hunting	
It is cruel to keep wolves in captivity.	Wölfe in Gefangenschaft zu halten ist grausam.
I would ban any kind of wild game hunting.	Ich würde es verbieten, Wildtiere zu jagen.
Killing wolves for fun is cruel.	Es ist grausam Wölfe zum Spaß zu töten.
In Germany, wolves’ abundance should increase.	In Deutschland sollte es mehr Wölfe geben.
